# Carbon-Based Nanomaterials Thin Film Deposited on a Flexible Substrate for Strain Sensing Application

**DOI:** 10.3390/s22135039

**Published:** 2022-07-04

**Authors:** Shiuh-Chuan Her, Yuan-Ming Liang

**Affiliations:** Department of Mechanical Engineering, Yuan Ze University, 135 Yuan-Tong Road, Chung-Li 320, Taoyuan 32003, Taiwan; s1065017@mail.yzu.edu.tw

**Keywords:** strain sensing, hybrid nanomaterials, piezoresistive behavior, gauge factor

## Abstract

Hybrid nanomaterial film consisting of multi-walled carbon nanotubes (MWCNT) and graphene nanoplatelet (GNP) were deposited on a highly flexible polyimide (PI) substrate using spray gun. The hybridization between 2-D GNP and 1-D MWCNT reduces stacking among the nanomaterials and produces a thin film with a porous structure. Carbon-based nanomaterials of MWCNT and GNP with high electrical conductivity can be employed to detect the deformation and damage for structural health monitoring. The strain sensing capability of carbon-based hybrid nanomaterial film was evaluated by its piezoresistive behavior, which correlates the change of electrical resistance with the applied strain through a tensile test. The effects of weight ratio between MWCNT and GNP and the total amount of hybrid nanomaterials on the strain sensitivity of the nanomaterial thin film were investigated. Experimental results showed that both the electrical conductivity and strain sensitivity of the hybrid nanomaterial film increased with the increase of the GNP contents. The gauge factor used to characterize the strain sensitivity of the nanomaterial film increased from 7.75 to 24 as the GNP weight ratio increased from 0 wt.% to 100 wt.%. In this work, a simple, low cost, and easy to implement deposition process was proposed to prepare a highly flexible nanomaterial film. A high strain sensitivity with gauge factor of 24 was achieved for the nanomaterial thin film.

## 1. Introduction

Flexible film sensors that are lightweight and have high sensitivity and durability have received tremendous attention in recent years. Increased demand for highly flexible and sensitive sensors have been observed for health care devices [1,2], soft robotic skin [3,4], and human motion detection [5,6]. Strain sensors have a wide range of applications, such as damage detection, structural health monitoring, and fatigue life prediction. Traditional strain sensors are fabricated using various metals and semiconductors [7,8]. These types of sensors are more fragile, with limited strain range [9]. The development of polymer nanocomposite-based strain sensors with high sensitivity and stretchability has attracted much attention over the decade. There are several different types of flexible strain sensors, including resistance [10], piezoelectric [11], capacitance [12], and inductance [13]. The resistance-type strain sensor generally consists of a conductive sensing element and flexible elastomers, which can convert the deformation into resistance change. Nanomaterials, such as carbon nanotubes (CNT) and graphene nanoplatelet (GNP), have been widely used as conductive sensing elements due to their excellent mechanical and electrical properties. Graphene is a single layer of carbon atoms with large specific surface area and excellent electrical conductivity. Its conductivity can be changed under applied strain. The piezoresistive behavior can be employed for strain sensing application [14]. CNT is one of the most promising materials for flexible strain sensors owing to their flexibility and remarkable electrical conductivity [15]. Moreover, polymers such as polydimethylsiloxane (PDMS) [16], polyurethane (PU) [17], polyimide (PI) [18], natural rubber [19], and epoxy [20] commonly serve as elastomer substrate for maintaining the nanofiller interfaces due to their excellent elasticity and high flexibility.

The polymer nanocomposite-based flexible sensor is generally prepared by incorporating the elastomer substrate with conductive nanomaterials. Li et al. [21] developed a flexible strain sensor based on MWCNTs/PDMS nanocomposites using oil-in-water Pickering emulsion method to detect human motion. Lv et al. [22] fabricated a graphene oxide/polypyrrole@polyurethane sponge pressure sensor. The GO/PPy@PU sponge sensor could detect a wide range of pressure 75 Pa–15 kPa with a high sensitivity of 0.79 ${\mathrm{KPa}}^{-1}$. Wang et al. [5] reported a graphene nanoplate/silicone rubber flexible tactile sensor with high sensitivities of 8.45 ${\mathrm{KPa}}^{-1}$ at 0–55 kPa and 195.02 ${\mathrm{KPa}}^{-1}$ at 55–80 kPa. Ahmad et al. [23] fabricated a MWCNTs/Alumina composite film gas sensor using a sol–gel synthesis technique to detect CO_2_. The effect of MWCNT concentration on the adsorption of CO_2_ was investigated. Larimi et al. [24] developed a wearable strain sensor which was prepared by infusing graphene nano-flakes into a rubber-like adhesive pad. The sensor was employed to monitor the knee movement, finger movement, and heartbeat.

In this work, a flexible nanomaterial strain sensor consisting of GNPs and MWCNTs was prepared through a gun spray on a polyimide (PI) substrate. The hybridization between 2-D GNP and 1-D MWCNT reduces the stacking among the nanomaterials and produces a thin film with a porous structure. The effects of nanomaterial content and the weight ratio between the GNP and MWCNT on the electrical resistance and strain sensitivity of the nanomaterial strain sensor were investigated.

## 2. Materials and Methods

### 2.1. Materials

MWCNT and GNP were used as conductive elements for the nanomaterial strain sensor due to their exceptional electrical conductivity, remarkable mechanical properties, and high aspect ratio. MWCNTs with average diameter and length of 8 nm and 200 μm, respectively, were bought from Conjutek Co., New Taipei City, Taiwan. GNPs with average thickness and lateral length of 3 nm and 5 μm, respectively, were purchased from Enerage Inc., Yilan County, Taiwan. Polyimide (PI) with a thickness of 0.05 mm and high flexibility was purchased from Lih-Kuang Industry Co., Ltd., Taichung City, Taiwan, and used as a substrate for the nanomaterial strain sensor.

### 2.2. Preparation of GNP and MWCNT Suspension

The GNP and MWCNT suspensions were prepared by adding GNP and MWCNT into deionized water with a concentration of 0.5 mg/mL. To enhance the dispersion of GNP and MWCNT, surfactant Triton X-100 was added to the deionized water with a concentration of 10 mg/mL prior to the incorporation of GNP and MWCNT. The surfactant Triton X-100 was dispersed in deionized water by a tip sonicator (Q700, Qsonica L.L.C., Newtown, CT, USA). The sonication process was conducted at a pulse mode with 10 s on and 20 s off for 30 min. After that, GNP and MWCNT were added to the solution and dispersed by a tip sonicator operated at a pulse mode for 2 h. Once the sonication process was completed, GNP and MWCNT were well dispersed in the suspension.

### 2.3. Fabrication of GNP and MWCNT Hybrid Nanomaterials Film

**Figure 1:**
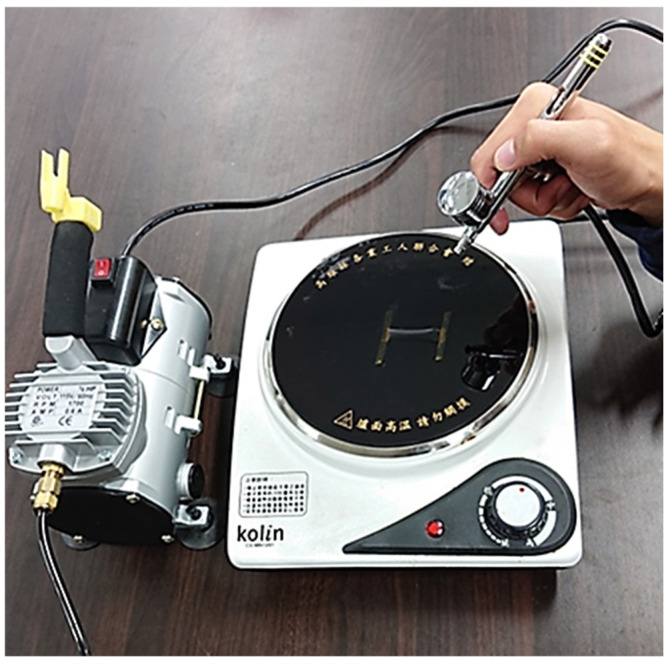
Experimental setup of gun spray deposition.

**Figure 2:**
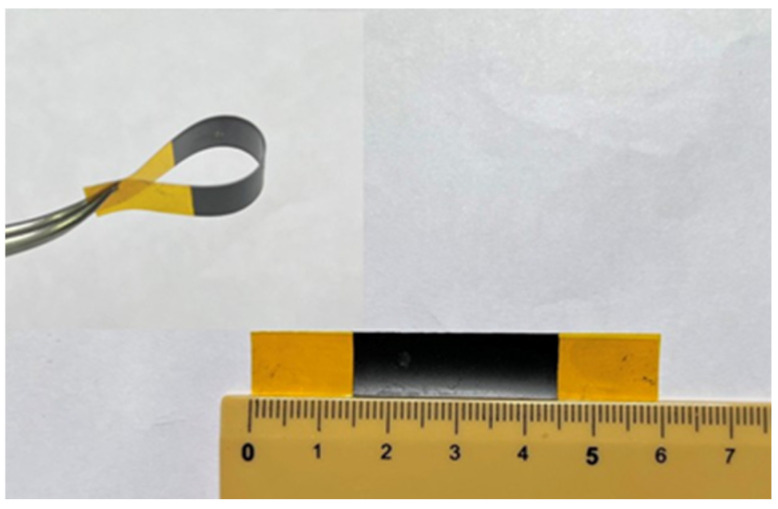
Flexible nanomaterial film deposited on a PI substrate.

Spray gun was used to deposit hybrid nanomaterials composed of GNP and MWCNT on a flexible polyimide (PI) substrate. Spray gun is considered to be a simple, low cost, and fast deposition process. A PI substrate with length 60 mm and width 10 mm was placed on a hot plate with a temperature of 100 °C. The well-dispersed GNP and MWCNT suspension was deposited on the PI substrate using spray gun, as shown in Figure 1. A spray gun with an atomizing nozzle diameter of 0.3 mm and pressure of 0.3 MPa was used to perform the deposition process. The thickness of the nanomaterial film can be controlled by the volume of the suspension sprayed deposition on the PI substrate. In this work, the concentration of hybrid nanomaterials (GNP and MWCNT) suspension was kept at a constant of 0.5 mg/mL, while the weight percentage of the GNP varied from 0% to 100%. The volume of hybrid nanomaterials suspension deposited on the PI substrate varied from 1 mL to 5 mL. The effects of the GNP content and total amount of hybrid nanomaterials (GNP and MWCNT) on the strain sensitivity of the hybrid nanomaterial film were investigated. A typical nanomaterial film on the PI substrate is shown in Figure 2. It demonstrates that entangled MWCNTs incorporated with GNPs introduce strong van der Waals forces between the MWCNTs and GNPs, resulting in a highly flexible nanomaterial film, as shown in Figure 2. The uniformity of the nanomaterial film prepared through a gun spray process can be affected by atomizing nozzle diameter, pressure, and the distance between the spray coater and the substrate. In this work, atomizing nozzle diameter, pressure, and the distance between the spray coater and the substrate were 0.3 mm, 0.3 MPa, and 15 mm, respectively. The film thickness was measured at five different locations to examine its uniformity. A good uniformity of the nanomaterials film thickness was achieved with a standard deviation less than 2%.

### 2.4. Characterization

The mechanical properties of the PI substrate including the Young’s modulus, tensile strength, and fracture strain were evaluated by a tensile test. The tensile tests were carried out on a universal testing machine (TPP S-200, Pin Tai Technology Co., Taichung City, Taiwan) equipped with a load cell of 200 N. The PI substrate was cut into a rectangular specimen with the length and width of 60 mm and 10 mm, respectively, and subjected to a tensile loading at a constant speed of 1 mm/min.

**Figure 3:**
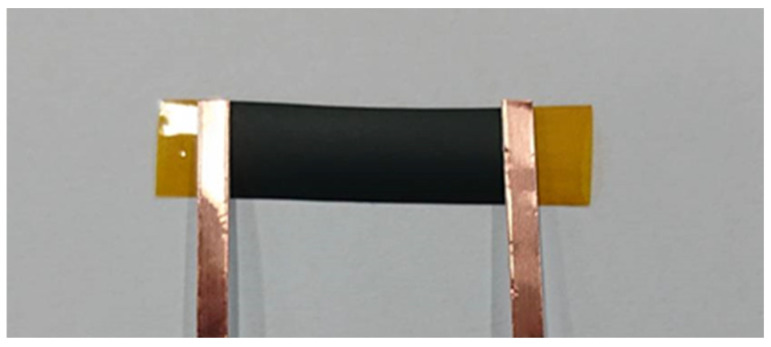
Strain sensitivity test specimen.

**Figure 4:**
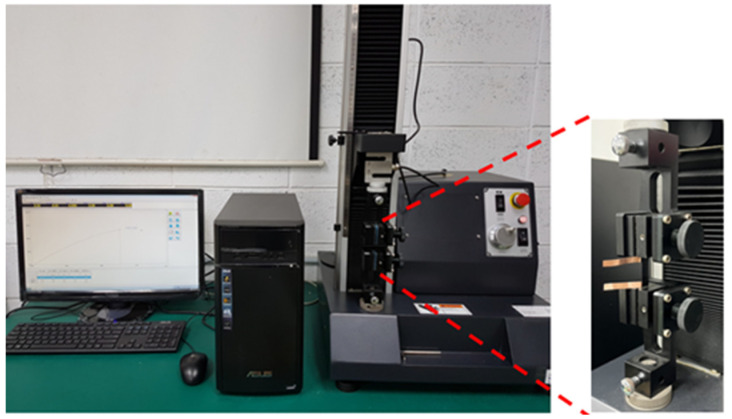
Experimental setup for the strain sensitivity test.

**Figure 5:**
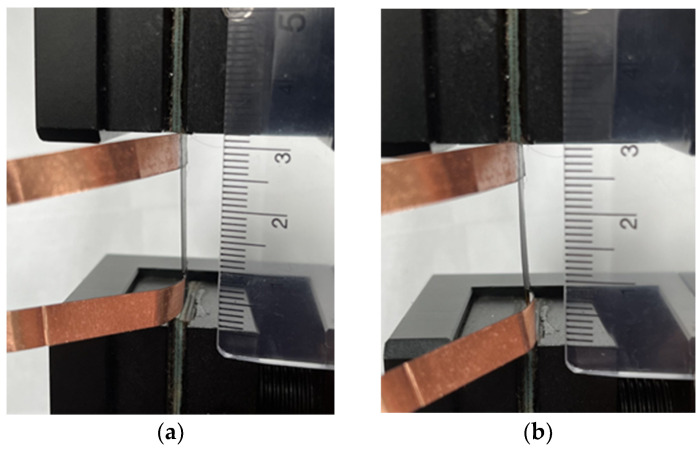
Nanomaterial film sensor subjected to a tensile test: (**a**) stretching 2 mm and (**b**) after stretch.

A rectangular nanomaterial film with length 30 mm and width 10 mm was deposited on the central area of a rectangular PI substrate with length 60 mm and width 10 mm. Two copper tapes were attached on the two ends of the nanomaterial film, respectively, to serve as electrodes, as shown in Figure 3. The test specimen was subjected to a tensile loading, as shown in Figure 4. Figure 5a,b illustrate the film sensor under 2 mm stretching and after stretch, respectively. The resistance change of the nanomaterial film in response to the applied strain of the tensile test was measured by a source meter (Keithley 2450, Beaverton, OR, USA). The strain sensitivity of the nanomaterial film is characterized by the gauge factor (GF), defined as follows.

(1) $$\mathrm{GF}=\frac{\Delta R}{R_{0}\varepsilon }$$

where $R_{0}$ denotes the resistance of the nanomaterial film at initial state, and ΔR represents the resistance change due to the applied strain ε.

## 3. Results and Discussions

### 3.1. Tensile Properties of PI

**Figure 6:**
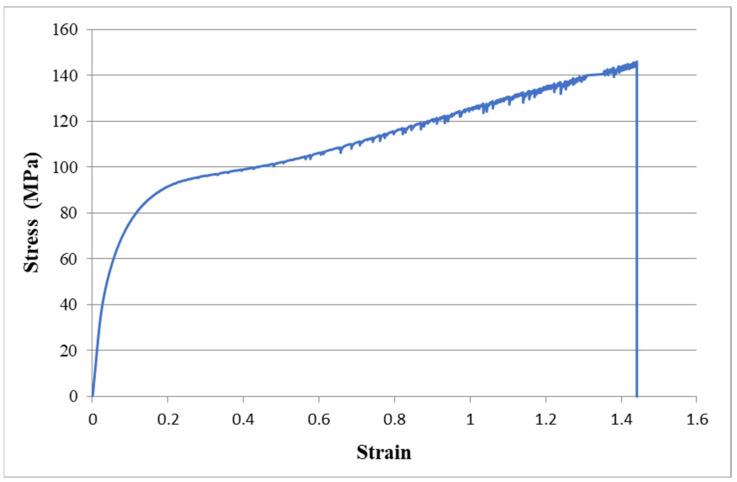
Stress–strain curve of PI substrate.

The tensile properties such as the Young’s modulus, tensile strength, and fracture strain can be extracted from the stress–strain curve of the tensile test. Figure 6 plots a typical stress vs. strain curve resulted from the tensile test for the PI substrate. In this work, three specimens were prepared and tested for the PI. The average and standard deviation of the tensile properties for the PI are presented in Table 1. It can be seen that the PI substrate exhibits a high stretchability of 146%.

### 3.2. Strain Sensitivity of Nanomaterial Film

**Figure 7:**
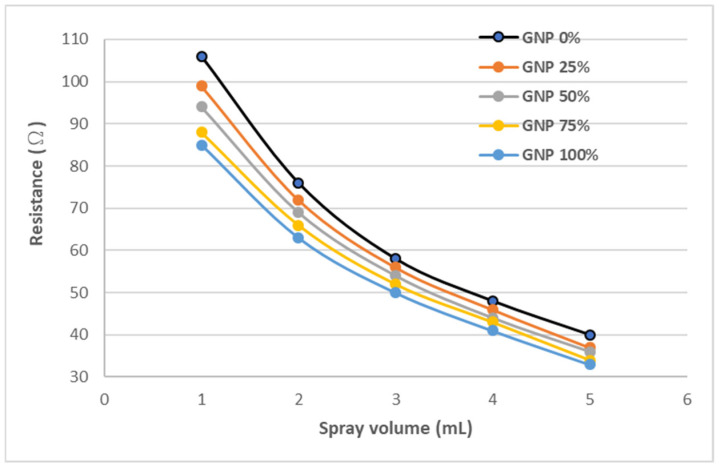
Resistance of hybrid nanomaterial film varying with spray volumes and GNP contents.

The electrical resistance of a nanomaterial film at initial state was measured by a source meter (Keithley 2450). In this work, the volume of the hybrid nanomaterials suspension sprayed on the PI substrate varied from 1 mL to 5 mL to investigate the effect of the total amount of the nanomaterials on the resistance of the nanomaterial film. In addition, the weight percentage of GNP varied from 0% to 100% for a specific volume of the hybrid nanomaterials suspension sprayed on the PI substrate, to study the influence of the GNP content on the resistance of the nanomaterial film. Table 2 lists the resistances of the nanomaterial films with various spray volumes and GNP contents. It can be observed that the resistance of the nanomaterial film decreased with the increase of both the spray volume and GNP content, as shown in Figure 7. This may be attributed to the higher electrical conductivity of GNPs in comparison with MWCNTs. Moreover, a significant Schotty barrier was exhibited between the GNPs and MWCNTs, resulting in an increase of the resistance as the MWCNTs content was increased.

**Figure 8:**
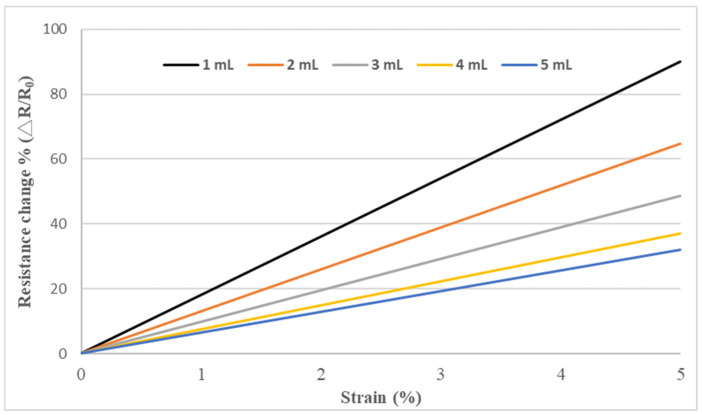
Resistance change vs. applied strain of the hybrid nanomaterial film with a fixed GNP content of 50 wt.% while the spray volume increased from 1 mL to 5 mL.

**Figure 9:**
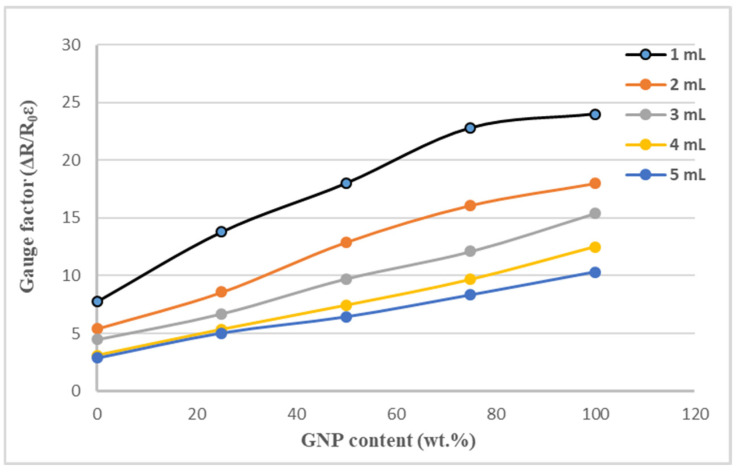
Gauge factor of hybrid nanomaterial film varying with spray volumes and GNP contents.

**Figure 10:**
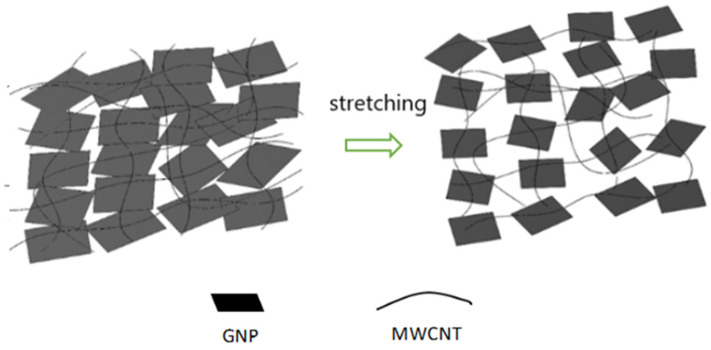
Conductive networks change of the hybrid nanomaterial film under stretching.

The test specimen, as shown in Figure 3, was placed in a universal test machine and subjected to a tensile loading. The electrical resistance of the nanocomposite film was measured by a source meter. The resistance changes of the nanomaterial film as a function of applied strain were recorded. Figure 8 plots the resistance change vs. applied strain of the nanomaterial films with spray volume varying from 1 mL to 5 mL on the PI substrate, while the GNP content was kept at a constant of 50 wt.%. The resistance change follows closely and monotonically with the applied strain. A good linearity between the resistance change and applied strain with the coefficient of determination greater than 0.99 depicts the feasibility of the nanomaterial film as a strain sensor. The slopes of the linear relationship between the resistance change and applied strain, as shown in Figure 8, for spray volumes of 1 mL, 2 mL, 3 mL, 4 mL, and 5 mL are 18, 12.9, 9.73, 7.43, and 6.43, respectively. The gauge factor, as defined in equation (1), can be deduced from the slope of the linear curve. Table 3 presents gauge factors of the nanomaterial films with various spray volumes and GNP contents. It appears that the gauge factor of the nanomaterial film increased with the increase of the GNP content and decreased with the increase of the spray volume, as shown in Figure 9. The piezoresistive effect for the nanomaterial strain sensor made from MWCNTs and GNPs is mainly attributed to the change of conductive networks in response to applied strain due to the loss of contact between the nanomaterials and tunneling effect in neighboring nanomaterials. A schematic diagram illustrated the change of conductive networks under stretching is presented in Figure 10. Contact resistance between the GNPs and MWCNTs resulting from the tunneling effect is the dominant factor for the variation of the resistance. With the increase of the strain, the tunneling distance will gradually increase, leading to an increase of the resistance. In this work, a variety of weight ratios between the GNP and MWCNT were included in a parametric study to investigate the influence of GNP and MWCNT on the strain sensitivity. It was found that GNP was more sensitive to the applied strain in comparison with MWCNT. In addition, the sensitivity of the proposed GNP film sensor was compared with the other strain sensors reported in the literatures, as shown in Table 4.

### 3.3. Experimental Measurement of Strain Using Nanomaterial Film Sensor

**Figure 11:**
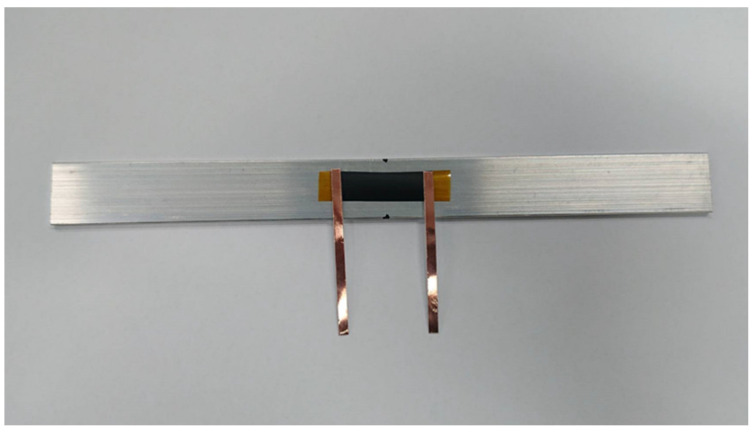
Nanomaterial film sensor bonded on an Al test specimen.

**Figure 12:**
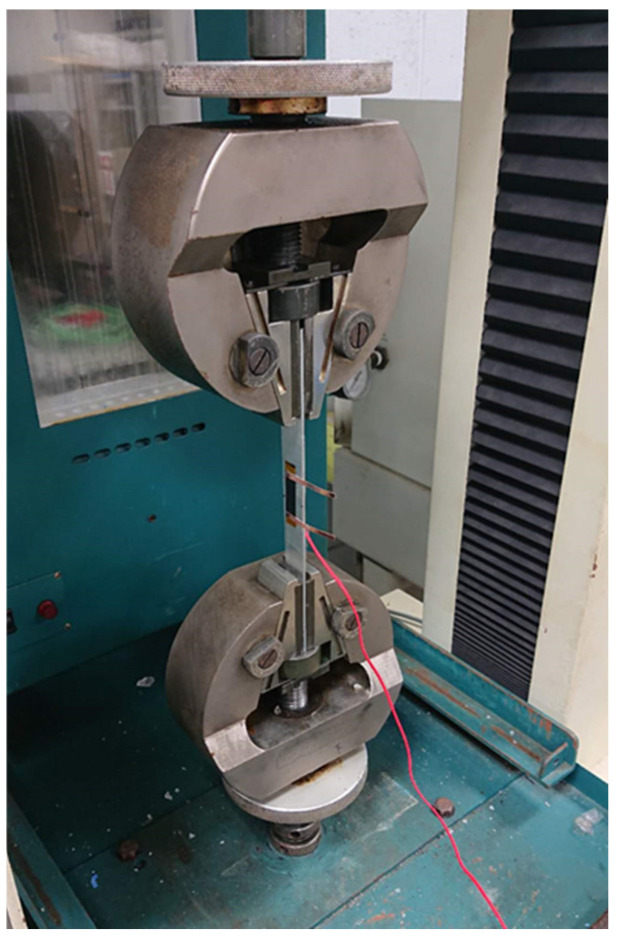
Al specimen subjected to tensile loading.

**Figure 13:**
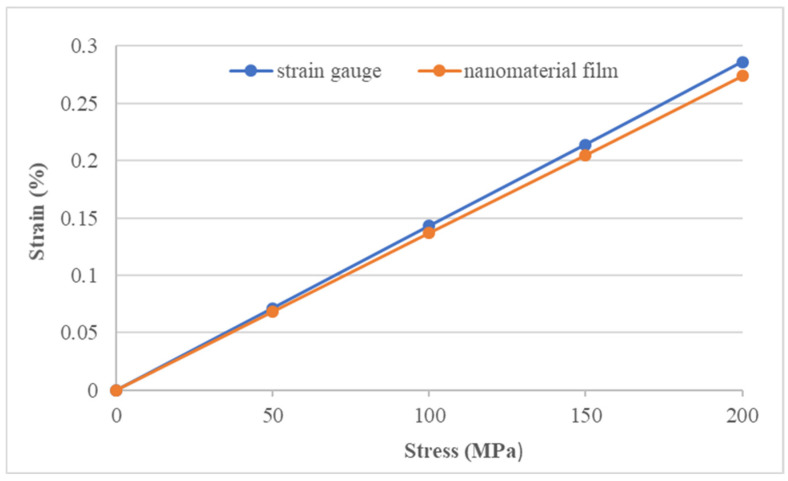
Strains of the Al specimen measured by the strain gauge and nanomaterial film sensor with strain sensitivity of 24.

**Figure 14:**
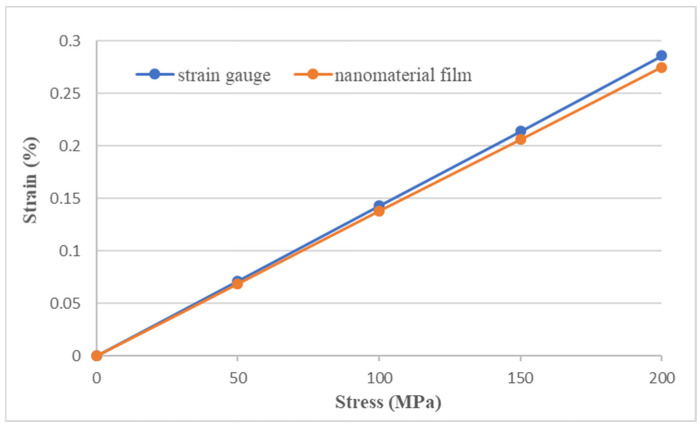
Strains of the Al specimen measured by the strain gauge and nanomaterial film sensor with strain sensitivity of 2.87.

A rectangular aluminum (Al) specimen with dimensions of 300 mm × 20 mm × 2 mm was served as a host structure. The nanomaterial film sensor was surface bonded on the central area of the Al specimen using epoxy resin, as shown in Figure 11. In addition, a strain gauge was adhered to the back side of the Al specimen to verify the strain measured by the nanomaterial film sensor. The Al specimen was subjected to a tensile loading using a universal machine (10 KS, Hounsfield, UK) at a loading rate of 5 mm/min, as shown in Figure 12. The nanomaterial film sensor, prepared by spraying 1 mL suspension with GNP content 100% on the PI substrate, exhibits the best strain sensitivity of 24 while the nanomaterial film obtained by spraying 5 mL suspension with MWCNT content 100% had the lowest strain sensitivity of 2.87, as shown in Table 3. These two nanomaterial film sensors were employed to measure the strain of the Al specimen. In the tensile test, the strain of the Al specimen measured by the nanomaterial film sensor was compared with the strain gauge. Figure 13 and Figure 14 plot the strains of the Al specimen measured by the nanomaterial film sensors with the strain sensitivity of 24 and 2.78, respectively. It can be seen that strain measured by the nanomaterial film sensor is in a good agreement with the strain gauge. The differences of the strain measured by the nanomaterial film sensors with stain sensitivity of 24 and 2.87 are 3.8% and 4.2%, respectively, in comparison with the strain gauge. This demonstrates that the nanomaterial film sensor was capable of measuring the strain with a good accuracy.

## 4. Conclusions

In this work, we reported the fabrication of hybrid (GNP and MWCNT) nanomaterials based flexible strain sensors and experimentally investigated their strain sensitivities. The hybrid nanomaterials film was deposited on a PI substrate using a spray gun. To ensure the uniform distribution of GNPs and MWCNTs in the spray gun process, horn sonication assisted with the surfactant Triton X-100 were used for the preparation of the nanomaterial film. The electromechanical property of the nanomaterial film was evaluated by piezoresistivity measurement, and the strain sensitivity was characterized using the gauge factor. A good linearity between the resistance change and applied strain over a wide range of strain illustrates the feasibility of the nanomaterial film to be used as a strain sensor. The influences of the weight ratio between GNPs and MWCNTs and the total amount of hybrid nanomaterials on the strain sensitivity of the nanomaterial film were investigated. It demonstrates that the gauge factor of the hybrid nanomaterial film can be tailored by varying the GNP content and total amount of the nanomaterials as it permits control of conductive network which directly correlates to the strain sensitivity. A high sensitivity of flexible nanomaterial film sensor was successfully prepared with a gauge factor of 24. In addition, the nanomaterial film sensor was verified by a strain gauge with an error of 4.2%. Present approach provides new insights into the fabrication and design of carbon-based nanomaterial strain sensor.

## Figures and Tables

**Table 1:** Tensile properties of the PI substrate.

Tensile Properties	PI Substrate
Young’s modulus GPa	1.19 ± 0.065
Tensile strength MPa	144 ± 0.90
Fracture strain	1.46 ± 0.018

**Table 2:** Resistances of hybrid nanomaterial films with various spray volumes and GNP contents.

	GNP	0%	25%	50%	75%	100%
Volume						
1 mL		106 Ω	76 Ω	58 Ω	48 Ω	40 Ω
2 mL		99 Ω	72 Ω	56 Ω	46 Ω	37 Ω
3 mL		94 Ω	69 Ω	54 Ω	44 Ω	36 Ω
4 mL		88 Ω	66 Ω	52 Ω	43 Ω	34 Ω
5 mL		85 Ω	63 Ω	50 Ω	41 Ω	33 Ω

**Table 3:** Gauge factors of hybrid nanomaterial films with various spray volumes and GNP contents.

	GNP	0%	25%	50%	75%	100%
Volume						
1 mL		7.75	13.78	18.0	22.8	24.0
2 mL		5.41	8.57	12.9	16.1	18.0
3 mL		4.47	6.70	9.73	12.1	15.4
4 mL		3.09	5.34	7.43	9.66	12.5
5 mL		2.87	5.03	6.43	8.32	10.3

**Table 4:** Comparison of gauge factors among the various strain sensors.

Sensor Type	Guage Factor
Buckypaper [25]	6.2
Epoxy/MWCNT [26]	0.43
Epoxy/GNP [27]	6.5
PDMS/MWCNT [28]	4
Metallic strain gauge [1]	2
GNP film [present]	24

## Data Availability

Data are available on request.
